# Correlation between changes in blood pressure with insulin resistance in type 2 diabetes mellitus with four weeks of pioglitazone therapy

**DOI:** 10.4103/0973-3930.45275

**Published:** 2008

**Authors:** Sandip Kumar Dash

**Affiliations:** Consultant in Neurology, Apollo Hospitals, Dhaka, Bangladesh. E-mail: drskdash@yahoo.co.in

Sir,

I have read the article by Hettihewa *et al*.[1] and found it to be very interesting. In this connection, I would like to mention that that in the present study, only two recordings of blood pressure were measured at an interval of 5 minutes, after 10 minutes of rest periods. It would have been better to monitor 24-hour ambulatory pressure measurement as it generates a more reproducible and clinically meaningful measurement than a single random cuff pressures. So it would have been better to monitor 24-hour pressure measurement so as to conclude the overall effect of the drug on blood pressure, including changes in nocturnal and diurnal blood pressure.

Secondly, weight gain is known to occur with all of the thiazolidinediones and is more in diabetic patients because of reduced glycosuria, sodium retention, and positive anabolic effects associated with improved glucose control. When used as monotherapy, the incidence of pedal edema ranges from 3% to 5% for each of the thiazolidinediones.[2] The incidence is greater when the drugs are used in combination with other glucose lowering agents. Edema was seen in 4.8% of patients on a pioglitazone monotherapy vs. 1.2% on placebo.[3] Vascular endothelial growth factor (VEGF) also has been implicated as a causal factor in thiazolidinedione-induced edema.[4] The increase in plasma volume related to thiazolidinediones may result from a reduction in renal excretion of sodium and an increase in sodium and free water retention and this fluid retention in turn can aggravate cardiac failure. In the present study, did any of the patients in the study group developed any adverse effect due to pioglitazone and any cardiovascular adverse effects? The reference (28) indicating mechanism of action in lowering blood pressure is not mentioned in references.

So while studying the blood pressure lowering effect of pioglitazone in type 2 diabetes mellitus, further study is needed regarding adverse events such as peripheral edema, weight gain along with any deterioration in cardiac function and the underlying mechanism of reduction in blood pressure.
